# Arterial blood pressure differences between AutoPulse™ and Lucas2™during mechanic cardiopulmonary resuscitation

**DOI:** 10.1186/s13049-016-0253-0

**Published:** 2016-04-30

**Authors:** Manuel Frey, Stefan Lötscher, Lorenz Theiler, Roland Albrecht

**Affiliations:** Department of Anaesthesia and Pain Therapy, University Hospital Inselspital, University of Bern, Bern, Switzerland; Swiss Air-Rescue, Rega-Center, P.O. Box 1414, CH-8058 Zurich, Switzerland

**Keywords:** Automated cardiopulmonary assist devices, Cardiopulmonary reanimation

## Abstract

We present a 39-year-old patient under constant mechanical CPR with an arterial line in place. The use of AutoPulse™ resulted in higher arterial pressures than the use of LUCAS2™.

## Findings

A 39-year-old patient, weight 82 kg, height 170 cm with a known coronary artery disease, who had undergone coronary artery revascularization and stenting of the left anterior descending coronary artery two years before, collapsed on admission to the emergency room. He was immediately resuscitated in-hospital, including rapid intubation and mechanical CPR using Lucas2 (Lund University Cardiopulmonary Assist Systems, Jolife AB, Lund, Sweden). Upon immediate admission to the cardiac catheter laboratory, an occlusion of the left main coronary artery was found and revascularization with stenting was established. However, return of spontaneous circulation (ROSC) was never achieved; the patient’s heart continuously showed pulseless electrical activity.

The cardiologists decided that considering the circumstances (no downtime, professional CPR beginning at the time of the cardiac arrest) and young age, the patient should be transferred to a hospital where extracorporeal membrane oxygenation (ECMO) could be performed.

**Fig. 1:**
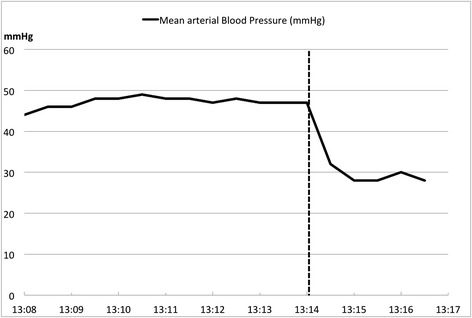
Mean arterial blood pressure (in mmHg). Vertical line at 13.14 h depicts time when AutoPulse was discontinued

When the HEMS crew met the patient he was under mechanical CPR with a Lucas2 correctly applied over the mid-to lower third of the sternum, as described in the instruction manual. Epinephrine was continuously applied at a rate of 13 micrograms per minute. The invasively monitored arterial pressure from the left radial artery showed a systolic pressure of 60 mmHg, endtidal CO_2_ was 3.0 kPa. Since the HEMS crew was using an AutoPulse device (ZOLL Circulation, San José, CA, USA), for transportation, the AutoPulse was placed at the same location as described in the instruction manual. With the AutoPulse in place, arterial pressure rose dramatically to 220 mmHg and stayed on that level for the remainder of the transfer (38 min). Even though there was no ROSC during that time, no additional medication was given. Endtidal CO_2_ level remained at a level comparable to the previously measured values (2.5 kPa). When arriving at the tertiary referring hospital a further change from AutoPulse to Lucas2 was necessary due to locally available equipment. Again, during the brief time necessary to change the devices, manual CPR was performed. A return of spontaneous circulation (ROSC) could never be established. The Lucas2 was placed at the correct spot again, since the pivot pressure point could still be seen. A distinct drop in arterial pressure could be noticed under CPR with Lucas2 from previously measured 220 mmHg to 60 mmHg under Lucas2 (see Fig. 1). Endtidal CO_2_ levels dropped by approximately 0.5 kPa in the following minutes.

ECMO was quickly installed, but unfortunately, one hour after start of ECMO, further therapy had to be stopped since the patient’s pH remained at 6.2; there was no myocardial contraction visible in the transesophageal echocardiography and the CT scan already showed signs of brain damage.

## Discussion

To our knowledge, this is the first report of the load-distributing band AutoPulse and the piston-driven system Lucas2 being used in the same patient while arterial blood pressure was continuously measured invasively by arterial cannulation. The change in blood pressure after exchanging the devices was impressive and is subject for discussion. The fact that both in the departing and in the arriving hospital the pressure was lower using Lucas2, while great emphasis was given on correct device placement, most likely rules out a misplacement of the device. Our findings corroborate an earlier technical report, which showed greater flow generated by AutoPulse [1]. In that study, which was supported by the manufacturer of the AutoPulse, deeper compression, longer depression time and stronger compression force were described as reasons for the higher peak power of AutoPulse. This could be a possible explanation for the higher incidence ROSC in the load-distributor group (odds 1.6 versus mechanical CPR) compared to the piston-driven group (no change versus mechanical CPR) found in a meta-analysis by Westfall et al. [2]. So far, only few studies comparing arterial pressure, coronary perfusion or myocardial perfusion were performed. Halperin et al. [3] demonstrated in a porcine model that the mean coronary perfusion pressure was 21 mmHg using AutoPulse compared to 14 mmHg in the manual CPR group. Timerman et al. showed in 2003 [4] that using AutoPulse under CPR resulted in increased aortic pressure (mean 153 mmHg versus 115 mmHg in the manual CPR group). Of note, all four studies mentioned were not fully independent from the manufacturers of AutoPulse. Consequently, in a recent meta-analysis by Li et al. [5], lower survival rates and no advantage of using mechanical compression devices were found for both in-hospital and out-of-hospital cardiac arrest patients. Additionally, in a retrospective analysis by Koga et al. [6], the use of AutoPulse was associated with higher rates of posterior rib fractures and abdominal injuries compared with manual chest compression.

As a final remark, our case report describes the difference in performance of two mechanical resuscitation devices; it does not focus on the overall medical treatment of the patient. Current ERC guidelines [7] do not recommend prolonged mechanical resuscitation in patients without ROSC despite successful coronary revascularization, and further high doses of epinephrine were not applied during transport. While the air transport of this patient reflects a desperate last-ditch attempt to safe a patient’s life, it is not backed by current guidelines.

## Conclusion

AutoPulse seemed to produce higher systolic and mean arterial pressures in this patient compared with Lucas2. The final reason for this observation remains unclear and further independent investigations are warranted.
